# Dead Teeth

**Published:** 1895-08

**Authors:** John G. Ranken


ARTICLE III.

### DEAD TEETH.

BY JOHN G. RANKEN, L. D. S., ENG.

**A paper read before the North of England Odontological Society.**

I have adopted the following classification of dead teeth:

1. After heroic extirpation of a live pulp;
2. After removing a devitalized pulp;
3. After removing a putrescent pulp;
4. When there is abscess with fistulous opening;
5. When there is abscess without fistulous opening, a blind abscess.

1. This classification includes all teeth requiring root treatment and filling.

2. The first step in the treatment of all dead teeth is to so shape the cavity in the tooth that easy access to the canals may be assured. In some cases it is advisable that an entirely new opening should be drilled, especially in the case of front teeth, and sometimes in that of back teeth with small interstitial cavities leading to an exposure.

The second step is the removal of the contents of the pulp chamber and canal, having special regard to the risks of forcing septic material through the apex of the canal and thus infecting or irritating apical tissues. A fine watchmaker's broach, a barbed Donaldson, an orange wood point forced up the canal—the pulp thus ejected whole—or some other kind of nerve extractor passed well to the apex and quickly rotated will often effect the removal of the pulp in an entire state; or by means of a Moray or a Gates-Glidden drill, taking special care to avoid the perforation or making of a false opening through the root, the canal may be thoroughly cleared of *debris.* Enlarge the orifice of the canal, inject absolute alcohol, and dry with a hot air-syringe or by passing a fine wire heated in the spirit lamp flame several times up and down. The canals will now be in a fit state to receive whatever kind of root-filling material may be most favored by the operator.

3. The first two divisions of my classification "after heroic extirpation of a live pulp" and "after removal of a devitalized pulp," are so nearly akin that we may generally fix upon the same treatment in either case. At no time, after careful removal of every fragment of the pulp out of each canal, and the thorough drying out of the canals by means of the injection of alcohol and the use of the hot air-syringe, will the roots be in a more aseptic condition and in a more fit state for the practice of immediate root-filling, the means by which this class of teeth are most often filled. It is as well to remember that the removal of all pulp tissue—so necessary an adjunct in the case of immediate root-filling—is, as regards the buccal roots of upper molars, the small roots of bicuspids and the anterior roots of lower molars a matter of no great certainty, and thus in such teeth immediate root-filling is not the infallible treatment it is claimed by many to be. Again, the objections to immediate root-filling after extirpation relates to the difficulties experienced of thoroughly drying the canals after hæmorrhage or any exudation which may have occurred during the operation, to the severe pain experienced by a patient during the forcing a filling to the apex of the canals, as is absolutely necessary for the root filling to be a success; and again the great soreness and discomfort experienced in some cases for many days after the immediate root-filling has been performed. Often these objections do not exist, or may be easily overcome or endured if it is desirable or necessary to take time into consideration.

4. The third class, or those dead teeth as they exist after the removal of a putrescent pulp, are often difficult to distinguish from teeth having blind abscess, and the avoidance of a tightly closed in dressing is, during the first stage of treatment, a desideratum, as, on account of the stimulation of treatment, the active formation of pus, when not present, is often instigated. Having cleared out the canals, the walls of which, in this class of tooth, are often soft and moist, and especially favorable to the careful usage of a Gates-Glidden, the method generally followed is to place loosely in each canal some strong antiseptic dressing on cotton wool, leave it for a week, and a state of quiesence having existed for that time, a permanent root-filling may be introduced with safety in nearly every case.

The antiseptics used for this kind of dressing are: Perchloride of Mercury, 1-250, Carbolic Acid, Eucalyptus, Oil of Cassia, Iodoform, and others specially favored by individual operators.

Although the immediate root-filler maintains that as the contents of the pulp chamber and canals are the immediate cause of an abscess, should it exist in such cases, and that owing to the minuteness of the apical foramen in many teeth it is impossible to secure proper drainage, therefore, if the apices of canals are filled with a permanent filling, the pent up pus will somehow cease to exist; the primary cause being removed, all careful operators will favor the slower method of treatment, with, in the vast majority of cases, a greater percentage of successes.

5. In the fourth class we have teeth abscessed and having a fistulous opening in connection with the so-called abscess sac. This fistulous opening is generally a suppurating tract, in many cases opening on the gums in the immediate vicinity of the offending tooth; in some cases the tract is of considerable length, and opens at a point some distance from its origin. The patient generally complains of a gumboil making its appearance periodically, enlarging, bursting, and subsiding after discharging its noxious contents into the mouth, no pain being felt, as a rule. This class of dead tooth, provided the abscess is not of very long standing, and absorption of the apices of the roots has not occurred to any very considerable extent, is amenable to immediate root-filling.

The best method of treatment is to clean out the canals as well as possible, pass a fine broach or Donaldson through the apex of the root, and then to place as far down the cleaned out canal as one can, without exerting any great pressure, the needle of a hypodermic syringe, pack around the needle gutta-percha, fill the syringe with carbolic acid, screw it on to the needle, and gentle pressure will almost immediately force a certain amount of the acid along the suppurating fistulous tract and produce a white eschar upon that surface of tissue where the gumboil has been in the habit of forming. Having accomplished this, healthy granulation is stimulated, throughout the tract, the canal may, after the withdrawal of the hypodermic needle and gutta-percha, be dehydrated with alcohol, dried with the hot air-syringe, and immediately filled, the main filling also may be inserted on the same occasion with almost perfect safety.

Another method, where the hypodermic syringe is not at hand, is to wrap around a fine broach cotton wool soaked in a strong antiseptic, and by a pumping motion force the antiseptic along the whole tract.

The objections to the immediate root filling of this class of teeth, are: First, it is better to find out whether our treatment is likely to be successful before inserting a permanent filling and secondly, the teeth are sometimes in such a septic condition that the surrounding dentinal tubuli are filled with the products of putrefaction, as is evidenced from the great change in colour. This state many operators affirm it is desirable to get rid of as far as possible by the continual changing of strong antiseptics in the canals, after dehydrating with alcohol and the use of the hot air syringe. I believe that little result of any kind is gained by the last mentioned method.

6. My last sub-division treats of that class of dead teeth which are generally the least amenable to conservative treatment. I speak of teeth having blind abscess. They are primarily difficult of diagnosis; we get them sometimes pointing, when the diagnosis is easy, and the treatment simplified by making an opening over the spot indicated by the pointing pus, after treating the tooth as one having a fistulous opening; at other times it is impossible even after passing a broach through the apex of the root canals to get a discharge of pus following the withdrawal of the broach, and yet circumstances in the history of the tooth may seem to indicate the presence of a blind abscess; yet it is urged upon us by many distinguished and successful practitioners that even this class of tooth is amenable to immediate root-filling. This class of tooth is the one in which immediate root-filling is most dangerous and unjustifiable. It must be remembered that, although we may find the canal or canals entirely free from obstructive material, yet the apical tissues are very often inflamed, although quiescent, and it is necessary that, for a successful issue to be obtained, these tissues should be got into such a state that, after the permanent closure of the root apex, no inflammation or collection of pus should be allowed to exist amongst the apical tissues as this might afterwards necessitate the removal of the tooth itself. In all cases of blind or suspected blind abscess, careful clearing of the canals—some operators even forcing a hole through the apical foramina until blood or pus drain down the canal—is of the utmost importance; leave the canals entirely free for a few days, and then loose antiseptic dressings on cotton wool should be inserted, and changed daily until they are withdrawn from the canals in a sweet and clean state.

If time is an object, the performance of rhizodontrophy is better by far than immediate root-filling, and in any case it is as well after filling the canals to paint over the roots of the tooth, using a camel-hair pencil, a mixture of double strength tincture of Iodine and Fleming's Tincture of Aconite.

In all cases the apical end of the root canals of the dead tooth should be permanently closed as soon as the canal is free from putrescent material, having of course allowed a sufficiency of time and correct course of treatment to have assured ourselves that the canals will remain clean.

The question arises, what is the best material with which to permanently close the root foramina so that no septic material may gain entrance into the root canal? That it should be antiseptic, easy of introduction—so as to be adaptable to the shape of the canal—and of such a nature as to reach the apex and yet be easy of removal should the necessity arise are the essentials of a root-filling.

Nearly every operator has his own pet root-filling, which he thinks is undoubtedly the best; some few of these I will very slightly touch upon. Gutta-percha points stand first in the field in answering most effectually the demands on a permanent root-filling. Three distinct methods of inserting them are employed in practice :—

I. The canal is thoroughly dried, mopped out with chloroform, and a suitable sized point driven carefully, with the aid of a root-filler, to the apex of the canal.

II. The canal being dried, a chloro-percha solution, which when thin seems as susceptible to capillary attraction as water, is forced into the canal about to be filled, then a suitable point is chosen and driven home, great care being exercised that none of the solution is forced through the apex, as disastrous results are likely to follow such a mishap.

III. A saturated solution of aristol in chloroform being worked up the canal in the same way as the chloro-percha solution and a G. P. point driven home; it is asserted by some operators that if any of the solution passes, as a result of pressure, through the apical foramen, no unfortunate result will follow, the aristol solution not being of an irritating nature.

Gold and copper were of a length and tapering fineness suitable to the canal to be filled, in conjunction with chloro percha, seems the next nearest to the ideal root-filling.

Gramme's copper canal points, in conjunction with bees'-wax, which by the aid of a hot bristle is first inserted into the canal, and, it is claimed, the dental fibrils also, seem to afford a large amount of satisfaction. The copper canal point having been slightly heated is forced into the wax, containing canal in much the same way as the G. P. points are forced into the canals containing chloro-percha solution.

Wood points soaked in a carbolized resin may be used in the same way as the metal points.

Celluloid strips having collodion as a conductor have recently been introduced by Mr. Tomes, and he claims that they make a most effective root-filling.

Bee's-wax passed into the canal in threads and a heated bristle passed up to melt the wax until the canal is filled, oxy-phosphate and oxy-chloride of zinc, plaster of Paris, and amalgam, of which Sullivan's on account of its antiseptic, preservative and strengthening action, is stated to be the best, all have their adherents.

Lastly, we have various dressings of wool, and such drugs as Iodoform, Eucalyptus, Oil of Cassia, Perchloride of Mercury (1-1,000), Carbolic and Creosote, which as dressings possess a large amount of merit, yet are not admissible as permanent root-fillings, although they are sometimes used as such.

I thank you gentlemen for your kind attention to what must have been, on account of its antiquity, somewhat dry matter to you, and hope that a severe criticism may be meted out upon my effort, in order that I may benefit by your matured experience.—*Dental Record.*

